# Association of gastric and duodenal ulcers with anthropometry and nutrients: Korean National Health and Nutrition Examination Survey (KNHANES II-IV) 2001-2009

**DOI:** 10.1371/journal.pone.0207373

**Published:** 2018-11-15

**Authors:** Bum Ju Lee, Jihye Kim, Keun Ho Kim

**Affiliations:** Future Medicine Division, Korea Institute of Oriental Medicine, Daejeon, Republic of Korea; University of Malaya Faculty of Medicine, MALAYSIA

## Abstract

**Objectives:**

The objective of this study was to examine the association of peptic ulcer disease (PUD), including gastric ulcer and duodenal ulcer, with obesity-related indices, nutrients, and blood parameters in Korean adults.

**Methods:**

Data were obtained from the Second-Fourth Korean National Health and Nutrition Examination Survey (KNHANES II-IV). Binary logistic regression was carried out to analyze the association between PUD and all variables in the crude analysis; in a subsequent analysis, adjustments were made for age, region, house type, number of snacks per day, and number of household members.

**Results:**

PUD exhibited the highest association with age in both men and women among all variables used in this study. In men, only body mass index was associated with PUD in both the crude and adjusted analyses. PUD was associated with weight, height, and fat in the crude analysis, but these associations disappeared after adjustment for confounders. Vitamin B2, hemoglobin, and glucose were related to PUD, but these associations became nonsignificant in the adjusted analysis. Water, vitamin C, and potassium were not associated with PUD in the crude analysis but were associated with PUD after adjustment for confounders. In women, systolic blood pressure and height were associated with PUD. PUD was also related to waist circumference, the waist-to-height ratio, fat, and cholesterol, but these associations became nonsignificant after adjustment for confounders. Vitamin C, protein, niacin, sodium, energy, vitamin B2, vitamin B1, and aspartate aminotransferases were associated with PUD in only the crude analysis. PUD was not associated with diastolic blood pressure, water, vitamin A, or glucose, but these factors were associated with the disease in the adjusted analysis.

**Conclusion:**

Older age was a risk factor for PUD in Korean adults, and the association of PUD with most nutrients and anthropometric indices may differ according to gender.

## Introduction

Peptic ulcer disease (PUD), including gastric ulcer and duodenal ulcer, is a common digestive disease with high mortality over the past centuries [1]. The prevalence of PUD is widely distributed throughout the world, including Denmark (5.6%) [2], Hong Kong (15%) [3], India (4.72%) [4], Shanghai population (17.2%) [5], South China (23%) and North China (9.7%) [3, 6], Iran (8.20%) [7], and Western populations (4.1%) [5]. PUD is associated with various risk factors, such as Helicobacter pylori (H. pylori) infection, aging, gender, education level, income, obesity and abdominal adiposity, nutrients, blood parameters, and lifestyle [5, 8–18].

To date, many studies have reported various risk factors for PUD. For example, a high body mass index (BMI) and current smoker or smoking period are independent indicators of asymptomatic PUD in Taiwan [19, 20], but alcohol intake is not associated with PUD in Japanese men in Hawaii [19]. High education level is negatively associated with PUD in Taiwan [20]. Gastrointestinal ulcers are highly related to age in the US [21]. Additionally, PUD is associated with dietary intake [13]. For example, dietary fiber intake was found to contribute to a decrease in the prevalence of PUD, esophageal cancer, colorectal cancer, gallbladder disease, constipation, and hemorrhoids [14, 15, 22–24].

The association of gastric and duodenal ulcers with adiposity is still controversial. Many studies have suggested a relationship between obesity and PUD [11, 20, 21], while several studies have reported that obesity and PUD have no relevance [25]. For example, high BMI is an independent indicator of PUD in Taiwan and the US [11, 20, 21], whereas subjects with PUD tend to have a low BMI in China [5]. The objective of this study was to investigate the association of PUD, including gastric ulcers and duodenal ulcers, with anthropometry and nutrients based on a recent Korean National Health and Nutrition Examination Survey (KNHANES) 2001–2009 in Korea. The findings of this study will provide clinical information to prevent and manage gastric and duodenal ulcers in Korean adults.

## Material and methods

### Subjects and definitions

In this retrospective cross-sectional study, data were obtained from KNHANES II-IV conducted by the Korea Centers for Disease Control and Prevention (KCDC) in 2001–2009 to study the health and nutrition status of the Korean population. The KNHANES is a statutory survey on the health behavior of people, the current status of chronic diseases, and the actual condition of food and nutrition consumption based on Article 16 of the National Health Promotion Act and the government-designated statistics (Approval No. 117002). The KNHANES was conducted every three years from the first KNHANES (1998) to the third KNHANES (1998), and it has been reorganized as a yearly survey system and has been conducted annually since the fourth KNHANES (2007–2009). The KNHANES II-IV were approved by the Korea Centers for Disease Control and Prevention (2007-02CON-04-P, 2008-04EXP-01-C, and 2009-01CON-03-2C) and were conducted in accordance with the Declaration of Helsinki. The present study was approved by the Institutional Review Board of the Korea Institute of Oriental Medicine for access and analysis of open source data from the KNHANES II-IV, with a waiver of documentation of informed consent (IRB No.I-1804/002-004).

**Fig 1:**
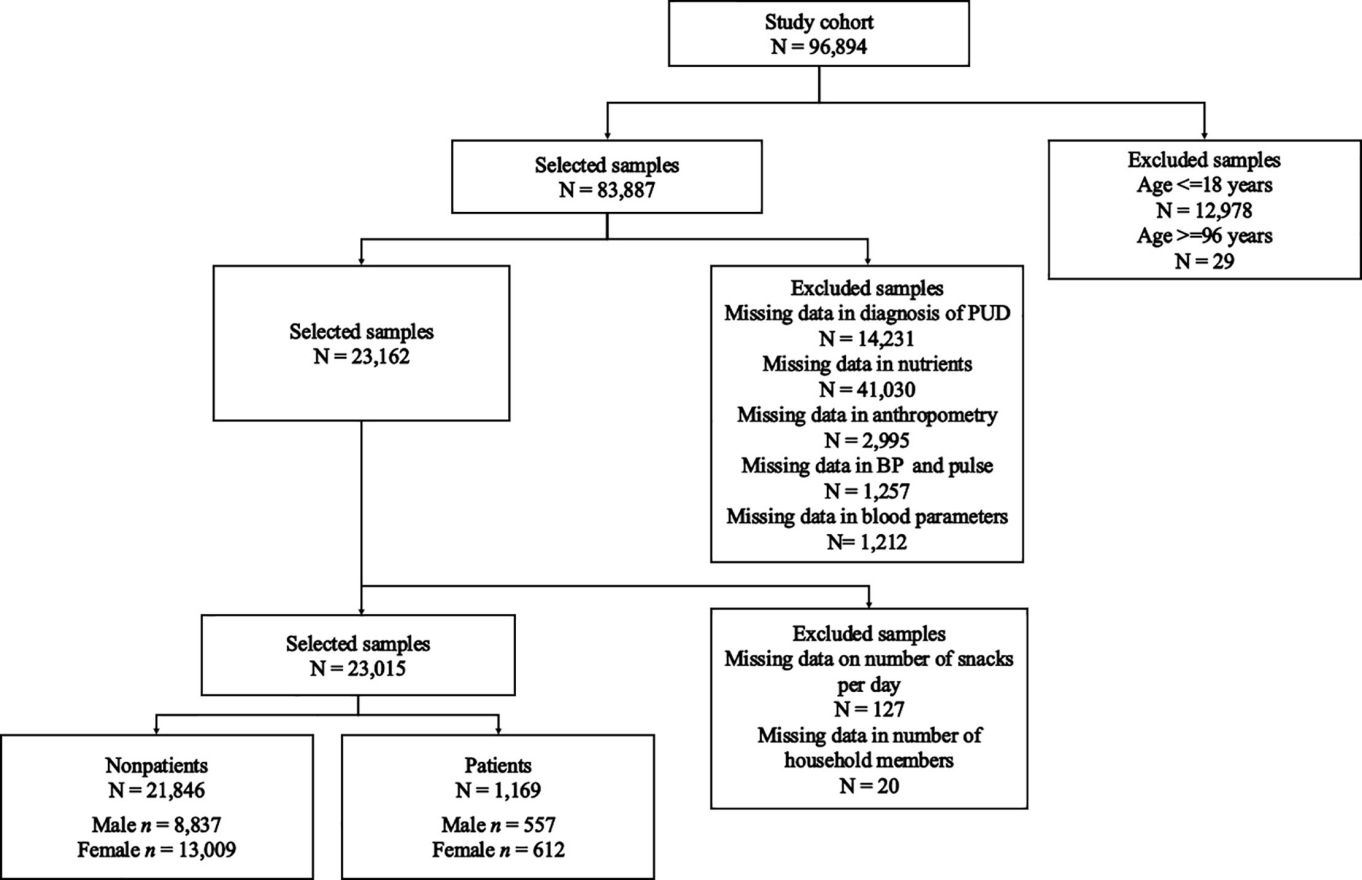
Sample selection procedure. PUD: peptic ulcer disease; BP: blood pressure.

The KNHANES II-IV included a total of 96,894 participants from 16 major cities in Korea, such as Seoul, Busan, Ulsan, Incheon, Daegu, Daejeon, Gwangju, Gangwon-do, Jeollabuk-do, Chungcheongbuk-do, Chungcheongnam-do, Gyeongsangnam-do, Jeollanam-do, Gyeongsangbuk-do, Gyeonggi-do, and Jeju Island. The KNHANES was not repeated in the same subjects. The rolling survey sampling method was applied to ensure independence and homogeneous characteristics among the selected samples. Thus, the data were not duplicated. Participants were selected based on inclusion and exclusion criteria. Of 96,894 subjects identified from the KNHANES II-IV (2001–2009) survey, we excluded those with 1) no information from a physician regarding PUD diagnosis; 2) no information on nutritional components (calcium, ash, fiber, etc.); 3) no information on vital signs and anthropometric indices, including height, weight and waist circumference (WC); or 4) missing data on blood parameters. As a result, a total of 23,015 Korean individuals (nonpatients = 21,846 and patients = 1,169) aged 19–95 years were eligible for the study. Written informed consent regarding the survey and blood analysis has been obtained from all subjects since 2001. The detailed inclusion and exclusion criteria and the number of subjects according to criteria are described in Fig 1.

Regarding the diagnosis of PUD, participants were classified as having PUD if they answered “Yes” to the question, “Do you have a physician-diagnosed peptic ulcer [26]?” Patients with a previous PUD who had fully recovered were not included in the analysis [26]. In the KNHANES II-IV, PUD was divided into 2 major subtypes: peptic ulcer and duodenal ulcer, which differed from the KNHANES I, which included gastritis, gastric ulcers, and duodenal ulcers.

### Measurements

In this study, the data consist of four types: demographic characteristics, anthropometric indices related to obesity, nutrients (dietary intake per day), and blood parameters. We considered demographic characteristics such as gender, systolic and diastolic blood pressures (SBP and DBP), house type, number of snacks per day, and number of household members. The health interview questionnaire collected information on medical conditions using a face-to-face interview method in the mobile examination center. Well-trained dietitians visited the homes of the subjects one week after the health interview and health examination and collected data related to the nutrition survey [27]. To determine whether there is an association between anthropometric measures and PUD, we examined weight, height, WC, the waist-to-height ratio (WHR), and BMI. A total of 11 nutrients, such as vitamins, protein, sodium, niacin, and fat, were tested in this study. The nutrition survey of KNHANES included diet information, such as 24-hour recall, dietary habits of participants, and a food frequency questionnaire [27]. All subjects reported all food and drinks consumed during the previous day in a face-to-face interview. The daily dietary intake was estimated using data from a single 24-hour recall form. The questionnaire on food intake was designed as an open-ended survey to report the consumption of various foods using the 24-hour recall method [28]. The daily dietary intake, such as water, vitamin A, vitamins B1 and B2, vitamin C, protein, niacin, sodium, potassium, fat and energy, was estimated based on the food items of KNHANES and was calculated using the Korean Foods and Nutrients Database of the Rural Development Administration [27, 29]. Blood parameters such as hemoglobin, glucose, triglyceride, high-density lipoprotein cholesterol (HDL), aspartate aminotransferases (AST), creatinine, and cholesterol were analyzed. The demographic characteristics and variables used in this study are described in Table 1.

**Table 1:** Basic characteristics of all variables evaluated in this study.

Variable		Men		Women	
		Normal	PUD	Normal	PUD
Subjects (No. of subject, %)		8837 (94.1%)	557 (5.9%)	13009 (95.5%)	612 (4.5%)
Age (Mean, SD)		47.38 (15.95)	54.89 (13.72)	47.23 (16.21)	55.53 (14.23)
SBP (mmHg)^†^		123.2 (16.49)	123.6 (17.47)	117.2 (18.74)	119.9 (19.32)
DBP (mmHg)^†^		79.94 (10.7)	79.05 (10.23)	74.45 (10.63)	75.02 (10.55)
Weight (kg)^†^		68.52 (10.67)	66.24 (9.94)	57.19 (8.854)	56.59 (8.706)
Height (cm)^†^		169.2 (6.716)	167.9 (6.284)	156.4 (6.551)	154.9 (6.458)
Waist circumference (cm)^†^		84.33 (8.754)	84.29 (8.753)	78.79 (9.805)	80.63 (9.477)
Waist-to-height ratio^†^		0.499 (0.053)	0.502 (0.053)	0.505 (0.069)	0.522 (0.067)
BMI (kg/m^2^)^†^		23.89 (3.115)	23.44 (3.037)	23.39 (3.396)	23.57 (3.284)
Water (g)^†^		1014 (679.1)	1008 (727.3)	780.5 (486.1)	748.7 (504.6)
Vitamin C (mg)^†^		117.5 (100.5)	119.8 (102.7)	110.4 (104)	100.3 (95.8)
Vitamin A (μgRE)^†^		868.5 (977.1)	878.8 (1245)	703.5 (759.2)	724.2 (689.8)
Protein (g)^†^		82.19 (42.57)	78.32 (37.81)	59.71 (31.67)	56.83 (31.9)
Niacin (mg)^†^		19.34 (10.81)	18.8 (12.55)	14.02 (7.99)	13.23 (8.179)
Sodium (mg)^†^		5977 (3274)	5821 (3501)	4446 (2890)	4195 (2793)
Potassium (mg)^†^		3326 (1561)	3363 (1561)	2686 (1385)	2611 (1350)
Fat (g)^†^		44.54 (35.45)	38.62 (30.93)	31.32 (25.44)	27.5 (27.25)
Energy (Kcal)^†^		2227 (882.2)	2159 (832.8)	1660 (659)	1602 (653.6)
Vitamin B2 (Riboflavin, mg)^†^		1.313 (0.808)	1.242 (0.74)	1.003 (0.652)	0.943 (0.671)
Vitamin B1 (Thiamine, mg)^†^		1.476 (0.896)	1.404 (0.752)	1.125 (0.68)	1.046 (0.69)
Triglyceride (mg/dl)^†^		156.1 (121.9)	162.7 (130)	117.3 (78.79)	121.4 (73.29)
HDL (mg/dl)^†^		44.44 (10.24)	44.34 (10.2)	49.05 (10.85)	48.59 (11.37)
Hemoglobin (g/dl)^†^		15.1 (1.195)	14.97 (1.342)	12.85 (1.189)	12.93 (1.158)
Glucose (mg/dl)^†^		98.67 (22.58)	100.9 (26.52)	95.57 (20.9)	95.76 (19.49)
Creatinine (mg/dl)^†^		1.014 (0.196)	1.014 (0.119)	1.002 (0.099)	1.005 (0.09)
Cholesterol (mg/dl) *		186.2 (34.92)	187.9 (33.99)	187.1 (36.01)	192 (35.59)
AST (IU/L)^†^		25.76 (16.63)	26.35 (15.82)	20.85 (9.241)	22 (8.524)
Number of household members		3.336 (1.298)	3.106 (1.244)	3.364 (1.373)	2.884 (1.387)
Region (city)					
	Seoul	1399 (15.8%)	49 (8.8%)	2162 (16.6%)	70 (11.4%)
	Busan	696 (7.9%)	47 (8.4%)	990 (7.6%)	49 (8.0%)
	Daegu	427 (4.8%)	33 (5.9%)	654 (5.0%)	34 (5.6%)
	Incheon	438 (5.0%)	25 (4.5%)	721 (5.5%)	29 (4.7%)
	Gwangju	336 (3.8%)	22 (3.9%)	471 (3.6%)	26 (4.2%)
	Daejeon	274 (3.1%)	23 (4.1%)	435 (3.3%)	16 (2.6%)
	Ulsan	206 (2.3%)	19 (3.4%)	311 (2.4%)	12 (2.0%)
	Gyeonggi-do	1628 (18.4%)	85 (15.3%)	2426 (18.6%)	79 (12.9%)
	Gangwon-do	357 (4.0%)	7 (1.3%)	467 (3.6%)	12 (2.0%)
	Chungcheongbuk-do	364 (4.1%)	23 (4.1%)	470 (3.6%)	37 (6.0%)
	Chungcheongnam-do	386 (4.4%)	34 (6.1%)	576 (4.4%)	46 (7.5%)
	Jeollabuk-do	405 (4.6%)	32 (5.7%)	582 (4.5%)	38 (6.2%)
	Jeollanam-do	467 (5.3%)	42 (7.5%)	628 (4.8%)	44 (7.2%)
	Gyeongsangbuk-do	657 (7.4%)	60 (10.8%)	934 (7.2%)	54 (8.8%)
	Gyeongsangnam-do	602 (6.8%)	43 (7.7%)	901 (6.9%)	45 (7.4%)
House type					
	Detached house	3521 (39.8%)	264 (47.4%)	4981 (38.3%)	300 (49.0%)
	Apartment	3605 (40.8%)	202 (36.3%)	5540 (42.6%)	224 (36.6%)
	Townhouse	613 (6.9%)	37 (6.6%)	937 (7.2%)	35 (5.7%)
	Multifamily housing	444 (5.0%)	28 (5.0%)	674 (5.2%)	25 (4.1%)
	House in business building	472 (5.3%)	20 (3.6%)	603 (4.6%)	22 (3.6%)
	Etc.	182 (2.1%)	6 (1.1%)	274 (2.1%)	6 (1.0%)
Number of snacks per day					
	Three times a day	600 (6.8%)	31 (5.6%)	1053 (8.1%)	35 (5.7%)
	Twice a day	1358 (15.4%)	78 (14.0%)	2851 (21.9%)	104 (17.0%)
	Once a day	3743 (42.4%)	218 (39.1%)	5626 (43.2%)	261 (42.6%)
	Rarely eats	3136 (35.5%)	230 (41.3%)	3479 (26.7%)	212 (34.6%)
Cancer (No. of subject, %)					
	Colon cancer	23 (0.3%)	2 (0.4%)	15 (0.1%)	1 (0.2%)
	Gastric cancer	55 (0.6%)	9 (1.6%)	42 (0.3%)	4 (0.7%)
	Liver cancer	15 (0.2%)	0 (0%)	1 (0%)	1 (0.2%)

* p < 0.05 and

^†^ < 0.0001 indicate significant differences between genders. The results were obtained by Student’s two-sample t-test. The data are presented as the mean ± standard deviation (SD) or as numbers of participants and percentages, N (%), for continuous or categorical variables, respectively. BMI: body mass index, PUD: peptic ulcer disease, HDL: high-density lipoprotein cholesterol, AST: aspartate aminotransferases. SBP: systolic blood pressure, and DBP: diastolic blood pressure.

### Statistical analysis

All statistical analyses were performed using SPSS 23 for Windows (SPSS Inc., Chicago, IL, US). Binary logistic regression was carried out to analyze the association between PUD and all variables in the crude analysis; subsequently, an analysis was conducted with adjustments for confounding factors such as age, region, house types, number of snacks per day, and number of household members. An independent two-sample t-test was performed to examine gender differences in baseline characteristics, as shown in Table 1.

## Results

Tables 2 and 3 list the association of PUD with anthropometric measures, blood parameters, and nutrients in men and women. In men, older age was most highly associated with PUD among all the variables used in this study (p < 0.001, odds ratio (OR) = 1.599 [1.466–1.743]). Among obesity-related indices, BMI was associated with PUD in the crude analysis (p = 0.001, OR = 0.862 [0.79–0.941]), and this association remained significant even after adjustment for potential confounders, including age, region, house types, number of snacks per day, and number of household members (adjusted p = 0.042, adjusted OR = 0.91 [0.832–0.997]). PUD was associated with weight (p < 0.001, OR = 0.798 [0.73–0.873]) and height (p < 0.001, OR = 0.833 [0.765–0.906]), but these associations disappeared after adjustments. Regarding nutrients, PUD was not associated with water and vitamin C in the crude analysis, but after adjustment for confounders, water (adjusted p = 0.023, adjusted OR = 1.098 [1.013–1.19]) and vitamin C (adjusted p = 0.025, adjusted OR = 1.098 [1.012–1.192]) were significantly associated with PUD. Fat was highly associated with PUD in the crude analysis (p < 0.001, OR = 0.812 [0.731–0.902]), but the association became nonsignificant in the adjusted analysis. Additionally, potassium was not related to PUD in the crude analysis but was associated with PUD in the adjusted analysis (adjusted p = 0.012, adjusted OR = 1.114 [1.025–1.212]). PUD was associated with protein (p = 0.036, OR = 0.906 [0.826–0.993]), vitamin B2 (p = 0.043, OR = 0.909 [0.829–0.997]), hemoglobin (p = 0.014, OR = 0.901 [0.829–0.979]), and glucose (p = 0.03, OR = 1.083 [1.008–1.164]) in the crude analysis, but these associations became nonsignificant in the adjusted analysis.

**Table 2:** Association of PUD with obesity indices, nutrients, and blood parameters in Korean men.

Variable	Crude		Adjusted	
	p	OR	p	OR
Age	<0.001	1.599 (1.466–1.743)	-	-
SBP	0.509	1.029 (0.945–1.12)	0.001	0.86 (0.786–0.941)
DBP	0.054	0.918 (0.842–1.001)	0.082	0.926 (0.849–1.01)
Weight	<0.001	0.798 (0.73–0.873)	0.241	0.944 (0.858–1.039)
Height	<0.001	0.833 (0.765–0.906)	0.125	1.082 (0.978–1.198)
Waist circumference	0.932	0.996 (0.914–1.085)	0.321	0.956 (0.876–1.044)
Waist-to-height ratio	0.160	1.063 (0.976–1.158)	0.112	0.929 (0.848–1.017)
BMI	0.001	0.862 (0.79–0.941)	0.042	0.91 (0.832–0.997)
Water	0.829	0.99 (0.908–1.08)	0.023	1.098 (1.013–1.19)
Vitamin C	0.588	1.023 (0.942–1.112)	0.025	1.098 (1.012–1.192)
Vitamin A	0.813	1.01 (0.93–1.097)	0.222	1.048 (0.972–1.13)
Protein	0.036	0.906 (0.826–0.993)	0.471	1.034 (0.944–1.134)
Niacin	0.260	0.949 (0.868–1.039)	0.162	1.064 (0.975–1.161)
Sodium	0.277	0.952 (0.871–1.04)	0.490	1.031 (0.945–1.124)
Potassium	0.584	1.024 (0.941–1.114)	0.012	1.114 (1.025–1.212)
Fat	<0.001	0.812 (0.731–0.902)	0.993	1 (0.904–1.105)
Energy	0.077	0.922 (0.843–1.009)	0.374	1.043 (0.951–1.145)
Vitamin B2 (riboflavin)	0.043	0.909 (0.829–0.997)	0.363	1.042 (0.953–1.14)
Vitamin B1 (thiamine)	0.062	0.915 (0.834–1.005)	0.449	1.036 (0.945–1.135)
Triglyceride	0.214	1.049 (0.973–1.131)	0.108	1.065 (0.986–1.151)
HDL	0.811	0.99 (0.908–1.078)	0.976	0.999 (0.918–1.087)
Hemoglobin	0.014	0.901 (0.829–0.979)	0.306	1.047 (0.959–1.143)
Glucose	0.030	1.083 (1.008–1.164)	0.710	1.015 (0.938–1.099)
Creatinine	0.969	1.002 (0.921–1.089)	0.529	0.966 (0.867–1.076)
Cholesterol	0.281	1.048 (0.963–1.141)	0.363	1.041 (0.955–1.134)
AST	0.417	1.031 (0.958–1.11)	0.789	1.011 (0.931–1.098)

Adjustment for age, region, house types, number of snacks per day, and number of household members. The results from the crude and adjusted analyses were performed by a binary logistic regression analysis.

**Table 3:** Association of PUD with obesity indices, nutrients, and blood parameters in Korean women.

Variable	Crude		Adjusted	
	p	OR	p	OR
Age	<0.001	1.646 (1.518–1.784)	-	-
SBP	<0.001	1.15 (1.065–1.241)	<0.001	0.818 (0.742–0.9)
DBP	0.192	1.055 (0.973–1.143)	0.041	0.915 (0.841–0.996)
Weight	0.101	0.933 (0.859–1.014)	0.914	1.005 (0.925–1.091)
Height	<0.001	0.801 (0.74–0.866)	0.012	1.138 (1.029–1.258)
Waist circumference	<0.001	1.2 (1.109–1.298)	0.910	1.005 (0.922–1.096)
Waist-to-height ratio	<0.001	1.262 (1.167–1.365)	0.481	0.967 (0.879–1.062)
BMI	0.187	1.055 (0.974–1.143)	0.268	0.953 (0.875–1.038)
Water	0.114	0.933 (0.856–1.017)	0.028	1.098 (1.01–1.193)
Vitamin C	0.019	0.893 (0.813–0.981)	0.668	0.98 (0.896–1.073)
Vitamin A	0.509	1.026 (0.951–1.108)	0.047	1.075 (1.001–1.154)
Protein	0.028	0.905 (0.828–0.989)	0.224	1.055 (0.968–1.149)
Niacin	0.017	0.896 (0.818–0.981)	0.424	1.036 (0.949–1.131)
Sodium	0.034	0.907 (0.829–0.993)	0.884	0.994 (0.912–1.083)
Potassium	0.190	0.945 (0.868–1.028)	0.321	1.043 (0.96–1.132)
Fat	<0.001	0.834 (0.756–0.919)	0.133	1.072 (0.979–1.174)
Energy	0.033	0.911 (0.836–0.992)	0.432	1.036 (0.949–1.131)
Vitamin B2 (riboflavin)	0.026	0.909 (0.836–0.989)	0.082	1.078 (0.991–1.173)
Vitamin B1 (thiamine)	0.005	0.879 (0.804–0.961)	0.793	1.012 (0.926–1.106)
Triglyceride	0.207	1.049 (0.974–1.13)	0.058	0.915 (0.834–1.003)
HDL	0.303	0.958 (0.882–1.04)	0.065	1.082 (0.995–1.176)
Hemoglobin	0.127	1.067 (0.982–1.16)	0.272	1.048 (0.964–1.139)
Glucose	0.824	1.009 (0.932–1.093)	0.014	0.885 (0.803–0.975)
Creatinine	0.513	1.022 (0.958–1.089)	0.950	1.002 (0.935–1.074)
Cholesterol	0.001	1.139 (1.054–1.232)	0.738	0.986 (0.906–1.073)
AST	0.003	1.089 (1.028–1.153)	0.954	1.002 (0.925–1.086)

Adjustment for age, region, house types, number of snacks per day, and number of household members. The results from the crude and adjusted analyses were performed by a binary logistic regression analysis.

In women, PUD had the strongest association with older age (p < 0.001, OR = 1.646 [1.518–1.784]) among all the variables used in this study, with a similar result found for men. SBP was associated with PUD in the crude analysis (p < 0.001, OR = 1.15 [1.065–1.241]) and remained the association in the adjusted analysis (adjusted p < 0.001, adjusted OR = 0.818 [0.742–0.9]). PUD was related to height in the crude (p < 0.001, OR = 0.801 [0.74–0.866]) and adjusted analysis (adjusted p = 0.012, adjusted OR = 1.138 [1.029–1.258]). WC (p < 0.001, OR = 1.2 [1.109–1.298]) and WHR (p < 0.001, OR = 1.262 [1.167–1.365]) had an association with PUD, but the association disappeared after adjustment for confounders. In nutrients, although protein, niacin, sodium, fat, energy, vitamin C, B2, and B1 were associated with PUD in the crude analysis, these associations became nonsignificant in the adjusted analysis. In contrast, Vitamin A was not associated with PUD in the crude analysis, but the association became significant after adjustment of confounders (adjusted p = 0.047, adjusted OR = 1.075 [1.001–1.154]). Cholesterol and AST were associated with the disease, but the associations disappeared after adjustment for age, region, house types, number of snacks per day, and number of household members. PUD was not related to glucose in the crude analysis, but in the adjusted analysis, glucose was associated with PUD (adjusted p = 0.014, adjusted OR = 0.885 [0.803–0.975]).

## Discussion

To date, many studies have reported that PUD is related to H. pylori infection, aging, gender, smoking, aspirin use, education level, income, obesity and abdominal adiposity, nutrients, blood parameters, and lifestyle [5, 8–18]. Everhart and Byrd-Holt [9] reported that aging, low education level, low income, and headache were risk factors for PUD in the United States. Wang and colleagues [20] suggested that people with a high education level had a lower association with PUD than those with a low education level in Taiwan. Kurata and colleagues [10] reported that the prevalence of PUD was higher in women than in men based on data from the International Classification of Diseases in the US Additionally, these researchers argued that hospitalizations for gastric ulcer treatment among women were notably increased in the 65-year-old age group. Li and colleagues [5] demonstrated that the prevalence of PUD was significantly higher in men than in women. Additionally, these researchers argued that subjects aged 40–49 years had a greater tendency to have PUD than those aged 30–39 years. Our findings are consistent with the results of previous studies [8, 9, 18, 22], indicating that older age is associated with PUD.

Regarding the relationship between obesity and PUD, the association of gastric and duodenal ulcers with adiposity is still controversial. Tsai and colleagues [25] examined the association of PUDs and BMI in three categories and found that BMI was not associated with gastric and duodenal ulcers in both men and women, whereas Boylan and colleagues [11] reported that BMI was associated with PUD in both the crude analysis and analysis adjusted for race, smoking, regular use of aspirin, alcohol intake, physical activity, and periodontal disease in US men. Additionally, these researchers argued that the WHR was associated with gastric ulcers but not duodenal ulcers. Kalichman and colleagues [12] documented that BMI, WC, the WHR, the skinfold index, and the fat-free mass index were associated with PUD and gastritis in central Russia, but the fat-free mass index was not related to PUD. Li and colleagues [5] demonstrated that the prevalence of PUD was higher in obese subjects than in normal subjects. Our findings indicated that BMI is related to PUD only in men and that a high BMI reduced the risk of PUD in men.

The Korean diet, which consists of a high intake of rice, soup, kimchi, and vegetables, includes relatively low amounts of proteins and lipids. Additionally, the diet is composed of relatively high amounts of carbohydrates, fiber, oxalate, and phytate compared with the Western diet [30]. Thus, the Western diet pattern is very different from the traditional Korean diet. Some studies have examined the association between nutrients and PUD. Elmståhl and colleagues [13] reported that intakes of energy, fiber, and fat (saturated, monounsaturated, polyunsaturated fats) were associated with PUD in Swedish subjects, while intakes of protein, calcium, and alcohol were not associated with PUD. Aldoori and colleagues [14] documented that insoluble and soluble fibers were risk factors for duodenal ulcers after adjustments for energy and age and that soluble fibers were more associated with duodenal ulcers than insoluble fibers in US male professionals, such as dentists, pharmacists, optometrists, and veterinarians. Additionally, these researchers argued that vitamins (B1, B2, E, and A) and potassium were inversely associated with duodenal ulcer, while vegetable and animal fats and total protein were not related to duodenal ulcer. Kearney and colleagues [15] suggested that protein, energy, and fat were not associated with nonulcer dyspepsia and PUD, but dietary fiber was associated with PUD and nonulcer dyspepsia. Ryan-Harshman [16] and colleagues mentioned that intake of fiber and vitamin A reduced the risk of duodenal ulcer and that there is little evidence that intake of fat, protein, or alcohol was associated with the risk of duodenal ulcer disease. Our findings are consistent with the results of a previous study [16], indicating that vitamin A is associated with PUD in women in the adjusted analysis, and agree with the results of previous studies [15, 16], indicating that fat, protein, and energy are not associated with PUD in both men and women in the adjusted analysis.

Regarding the association of blood parameters with PUD, Ifeanyi and colleagues [17] examined the difference in blood parameters between patients with PUD and nonpatients and suggested that only fasting blood sugar was significantly decreased in PUD patients compared with nonpatients. In addition, these researchers showed that HB, total cholesterol, triglyceride, HDL and LDL were not associated with PUD. Our findings agreed with the results of the study [17], indicating that HB, triglyceride, and HDL cholesterol are not associated with PUD in both the crude and adjusted analyses.

Eradication of H. pylori tends to increase total cholesterol, triglyceride, and BMI and increase the incidence of obesity, hypertriglyceridemia, and hypercholesterolemia in patients with PUD [31]. Subjects with H. pylori infection showed less favorable metabolic panels than those without H. pylori infection in a Chinese population [32]. Additionally, a high BMI was associated with the prevalence of H. pylori infection, indicating that subjects with obesity had a higher prevalence of infection than those without obesity [32]. Unfortunately, information about H. pylori infection was not considered in the KNHANES data; thus, we cannot discuss H. pylori infection and PUD in this study.

Previously, we reported an association of PUD with nutritional components and obesity based on KNHANES I [18]. As shown by both our previous study [18] and the present study, age is very highly associated with PUD in both men and women. Some findings were consistent with previous studies and the present study, but several findings were not consistent between the two studies. For example, the WC and weight-to-height ratio in men was not associated with PUD in either the previous study or the present study, while triglyceride was associated with PUD in the previous study but not in the present study. We assume that these differences between two studies were due to different study samples, diagnosis criteria of PUD, and confounders. Indeed, the two studies were based on different data: KNHANES I (1998) in a previous study [18], and KNHANES II-IV (2001–2009) in this study. Additionally, in KNHANES I (1998), the definition of PUD included gastritis, gastric ulcers, and duodenal ulcers. In contrast, the definition of PUD in KNHANES II-IV (2001–2009) included only gastric and duodenal ulcers, except for gastritis.

The present study has some limitations. The first limitation is the manner in which PUD was diagnosed, which was through the question “Do you have physician-diagnosed PUD?” in a self-reported questionnaire. To overcome this limitation associated with the response quality, only subjects with physician-diagnosed PUD were enrolled in this study. Second, the cross-sectional nature of the present study does not allow causal inference between PUD and the risk factor evaluated in this study. We cannot guarantee that our results are the same or similar to those of other countries, as the incidence of PUD and the characteristics of lifestyle, dietary, obesity, socioeconomic status, and climatic conditions differ according to countries and ethnic groups. Additionally, in this study, the excluded samples tended to be from younger patients. For example, the mean ages (standard deviation) of the patients whose samples were excluded were 31.19 (20.81; men) and 33.48 (22.09; women). The mean ages of patients whose samples were included were 47.84 (15.92; men) and 47.60 (16.22; women). Thus, our findings may not be representative of the Korean population.

## Conclusion

Gastric and duodenal ulcers are common digestive diseases throughout the world. In the present study, we demonstrated an association of PUD with nutrients and anthropometric indices and blood parameters in Korean adults and suggested that older age is one of the risk factors for PUD and that the association of PUD with nutrients and anthropometric indices differs according to gender. The findings of the present study provide clinical information to support the prevention and management of gastric and duodenal ulcers in Korean adults.
